# Fibromyalgia and microglial TNF-α: Translational research using human blood induced microglia-like cells

**DOI:** 10.1038/s41598-017-11506-4

**Published:** 2017-09-19

**Authors:** Masahiro Ohgidani, Takahiro A. Kato, Masako Hosoi, Makoto Tsuda, Kohei Hayakawa, Chie Hayaki, Rie Iwaki, Noriaki Sagata, Ryota Hashimoto, Kazuhide Inoue, Nobuyuki Sudo, Shigenobu Kanba

**Affiliations:** 1 Department of Neuropsychiatry, Graduate School of Medical Sciences, Kyushu University, Maidashi 3-1-1, Higashi-ku, Fukuoka 812-8582 Japan; 2 Department of Psychosomatic Medicine, Kyushu University Hospital, Maidashi 3-1-1, Higashi-ku, Fukuoka 812-8582 Japan; 3 Department of Life Innovation, Graduate School of Pharmaceutical Sciences, Kyushu University, Maidashi 3-1-1, Higashi-ku, Fukuoka 812-8582 Japan; 4 Department of Psychosomatic Medicine, Graduate School of Medical Sciences, Kyushu University, Maidashi 3-1-1, Higashi-ku, Fukuoka 812-8582 Japan; 5 Research Center for Children’s Mental Development, United Graduate School of Child Development, Osaka University, Yamadaoka 1-1, Suita, Osaka 565-0871 Japan

## Abstract

Fibromyalgia is a refractory disease characterized by chronic intractable pain and psychological suffering, the cause of which has not yet been elucidated due to its complex pathology. Activation of immune cells in the brain called microglia has attracted attention as a potential underlying pathological mechanism in chronic pain. Until recently, however, technological and ethical considerations have limited the ability to conduct research using human microglia. To overcome this limitation, we have recently developed a technique to create human-induced microglia-like (iMG) cells from human peripheral blood monocytes. In this study, we created the iMG cells from 14 patients with fibromyalgia and 10 healthy individuals, and compared the activation of iMG cells between two groups at the cellular level. The expression of tumor necrosis factor (TNF)-α at mRNA and protein levels significantly increased in ATP-stimulated iMG cells from patients with fibromyalgia compared to cells from healthy individuals. Interestingly, there was a moderate correlation between ATP-induced upregulation of TNF-α expression and clinical parameters of subjective pain and other mental manifestations of fibromyalgia. These findings suggest that microglia in patients with fibromyalgia are hypersensitive to ATP. TNF-α from microglia may be a key factor underlying the complex pathology of fibromyalgia.

## Introduction

Fibromyalgia, a representative form of non-organic pain, is a chronic disease that causes severe systemic pain with psychological suffering, resulting in disability and a lowered quality of life. Its clinical picture has long been documented; yet, fibromyalgia remains a refractory disease of unknown etiology to this day^1^. In a clinical study using fMRI, patients with fibromyalgia showed hyper-responsiveness to stimuli compared with healthy participants^2^. In addition, recent reports suggest that neuroinflammation caused by immune cells and inflammatory cytokines is related to the pathophysiology of fibromyalgia^3–5^. Fibromyalgia is suggested to be caused by complex bio-psycho-social factors with the central nervous system (CNS) as the pathological base.

**Figure 1:**
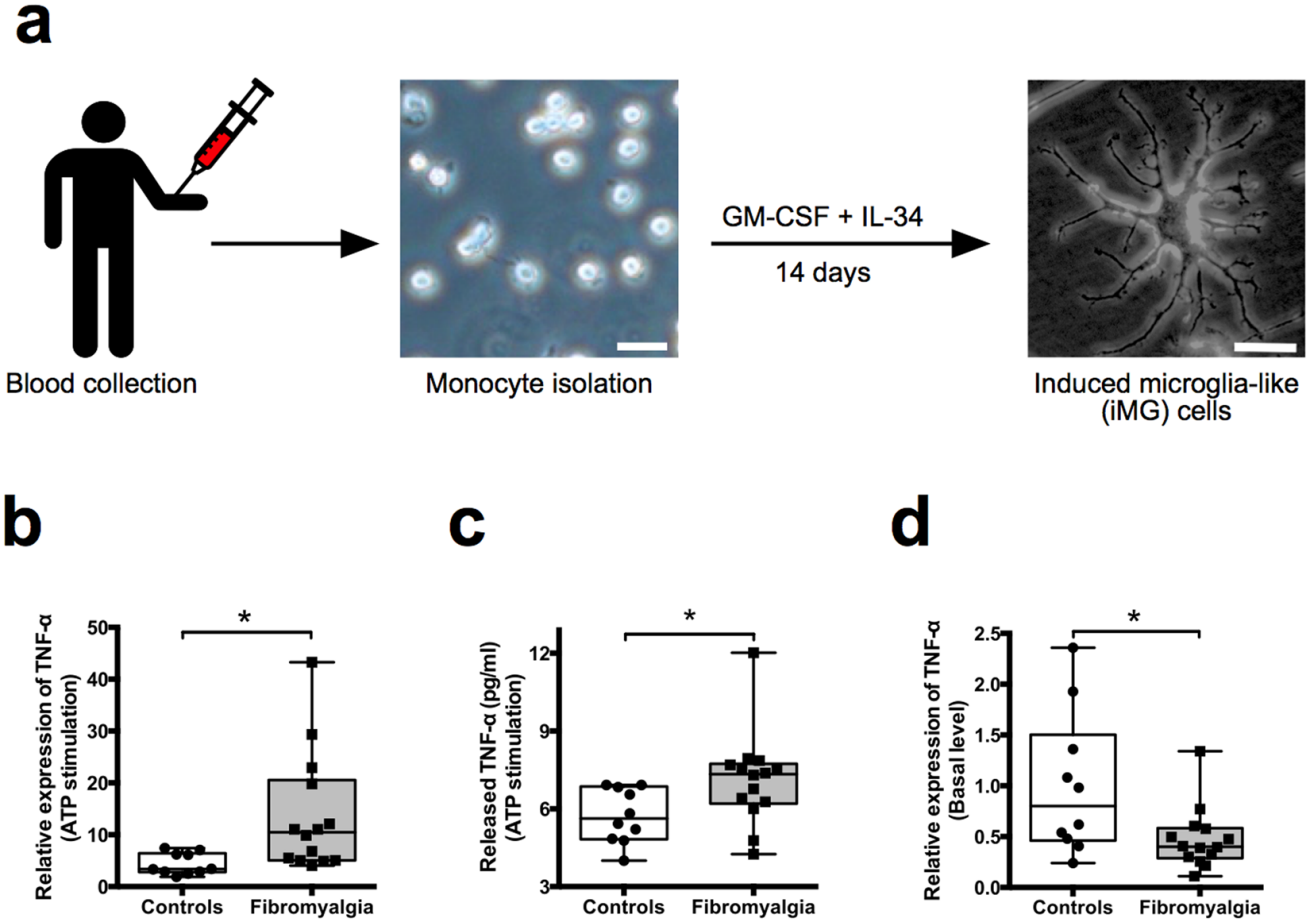
Human induced microglia-like (iMG) cells and gene expression of TNF-α during ATP stimulation. (**a**) Human induced microglia-like (iMG) cells. Scalebar, 50 μm. (**b**) Box-and-whisker plot showing gene expression of TNF-α during ATP stimulation in iMG cells from patients with fibromyalgia (n = 14; 25^th^ percentile, 5.08; mean, 13.6; 75^th^ percentile, 20.58) and healthy volunteers as a control group (n = 10; 25^th^ percentile, 2.81; mean, 4.39; 75^th^ percentile, 6.45). (**c**) TNF-α concentration of the supernatant released during ATP stimulation by iMG cells from patients with fibromyalgia (n = 14; 25^th^ percentile, 6.20; mean, 7.13; 75^th^ percentile, 7.74) and healthy volunteers (n = 10; 25^th^ percentile, 4.83; mean, 5.73; 75^th^ percentile, 6.86). (**d**) Basal gene expression of TNF-α in iMG cells from patients with fibromyalgia (n = 14; 25^th^ percentile, 0.29; mean, 0.48; 75^th^ percentile, 0.59) and healthy volunteers (n = 10; 25^th^ percentile, 0.46; mean, 1.00; 75^th^ percentile, 1.50).The y-axis represents the expression levels for each group normalized by the data of the non-ATP treatment group (NT: iMG cells without ATP stimulation) (**a**) or normalized by the data of healthy volunteers (**b**). As a result of Shapiro-Wilk normality test, statistical differences between groups were analyzed by Student’s *t*-test (two-tailed) (**a** and **c**) or Mann-Whitney *U* test (two-tailed) (**b**). *P < 0.05.

Microglia are immune cells in the CNS, and known to have inflammatory functions via releasing proinflammatory cytokines such as tumor necrosis factor (TNF)-α and interleukin (IL)-1β^6^. In rodent studies, we have reported the abnormalities of microglia as the pathological basis of chronic pain^7,8^. These rodent studies have indicated the over-activation of microglial cells in patients with chronic pain, however clinical molecular data are lacking due to ethical and technical issues. Thus, techniques to develop human microglia-like cells from non-brain tissues have been warrented^9^. Just recently, a technique to induce microglia-like cells from human pluripotent stem (iPS) cells has been reported^10–12^. The iPS technology enables cell-based assays in many fields such as embryology, pharmacology, and regenerative medicine; however, the iPS technology has some limitations for the modeling of non-genetic disease and needs to have much amount of time and costs^13^. On the other hand, we have recently developed a novel technique to induce microglia-like (iMG) cells directly from human peripheral blood (monocytes) by applying only two cytokines IL-34 and granulocyte macrophage colony-stimulating factor (GM-CSF) within two weeks without any gene modificaitons (Fig. 1a)^14,15^. Compared to the iPS cells, our iMG technique is simpler and has advantages of time and cost management. Another merit is that we can produce iMG cells without any gene modification. Fresh blood is needed to produce iMG cells, and we can not stock iMG cells in the present stage, which is major limitations of our iMG technique. We have already confirmed abnormalities in cellular responses of iMG cells derived from patients with Nasu-Hakola disease, which is a known primary microglia disease^14^. Furthermore, we have analyzed the gene expression paterns of iMG cells in both manic and depressive state of patients with bipolar disorders, and revealed state-dependent microglial experession patterns^16^. More recently, gene expression analysis from other research group has revealed that iMG cells show the most similar characteristics with primary human microglia in comparison with other cell types such as immortalized human microglia (SV40) and human macrophage^17^. Our previous studies and this recent report strongly suggest that iMG technique is a powerful tool for analyzing dynamic molecular pathophysiologies of microglia in not only genetic diseases but also non-organic diseases (including fibromyalgia and majority of psychiatric disorders).

Here, we created iMG cells from both healthy volunteers as healthy control group (HC) and patients with fibromyalgia (Supplementary Tables 1 and 2) to test the hypothesis that microglia are hyperactive in patients with fibromyalgia.

Extracellular ATP is known to function as a neurotransmitter and/or neuromodulator in the CNS and to modulate various physiological functions of microglia^18^. We previously reported a relationship between chronic pain and ATP in animal models^7^, and ATP has been implicated in the chronic pain mechanism^19^. We thus compared ATP-evoked responses of iMG cells from healthy volunteers and patients with fibromyalgia. Interestingly, gene expression of TNF-α is significantly higher in iMG cells from patients with fibromyalgia (Fig. 1b). In addition, TNF-α protein levels are also significantly higher in the culture supernatant of iMG cells from patients with fibromyalgia (Fig. 1c). In contrast, there are no statistically significant differences in the gene expression of IL-1β (Supplementary Fig. 1a,b). However, protein levels of IL-1β are significantly higher in the culture supernatant of iMG cells from patients with fibromyalgia (Supplementary Fig. 1c). In addition, There are no significant differences between two groups in secretion of IL-6 and IL-8, pro-inflammatory cytokines and IL-10, an anti-inflammatory cytokine (Supplementary Fig. 1d–f). At basal level, TNF-α gene expression levels are significantl lower in iMG cells from patients with fibromyalgia (Fig. 1d). On the other hand, there are no significant differences in phagocytic activity and gene expression of TNF-α and IL-1β during phagocytosis between healthy volunteers and patients with fibromyalgia (Supplementary Fig. 2). These results suggest that microglia in patients with fibromyalgia are hyperresponsive to ATP stimulation, which may result in increasing the release of TNF-α in the CNS.

**Figure 2:**
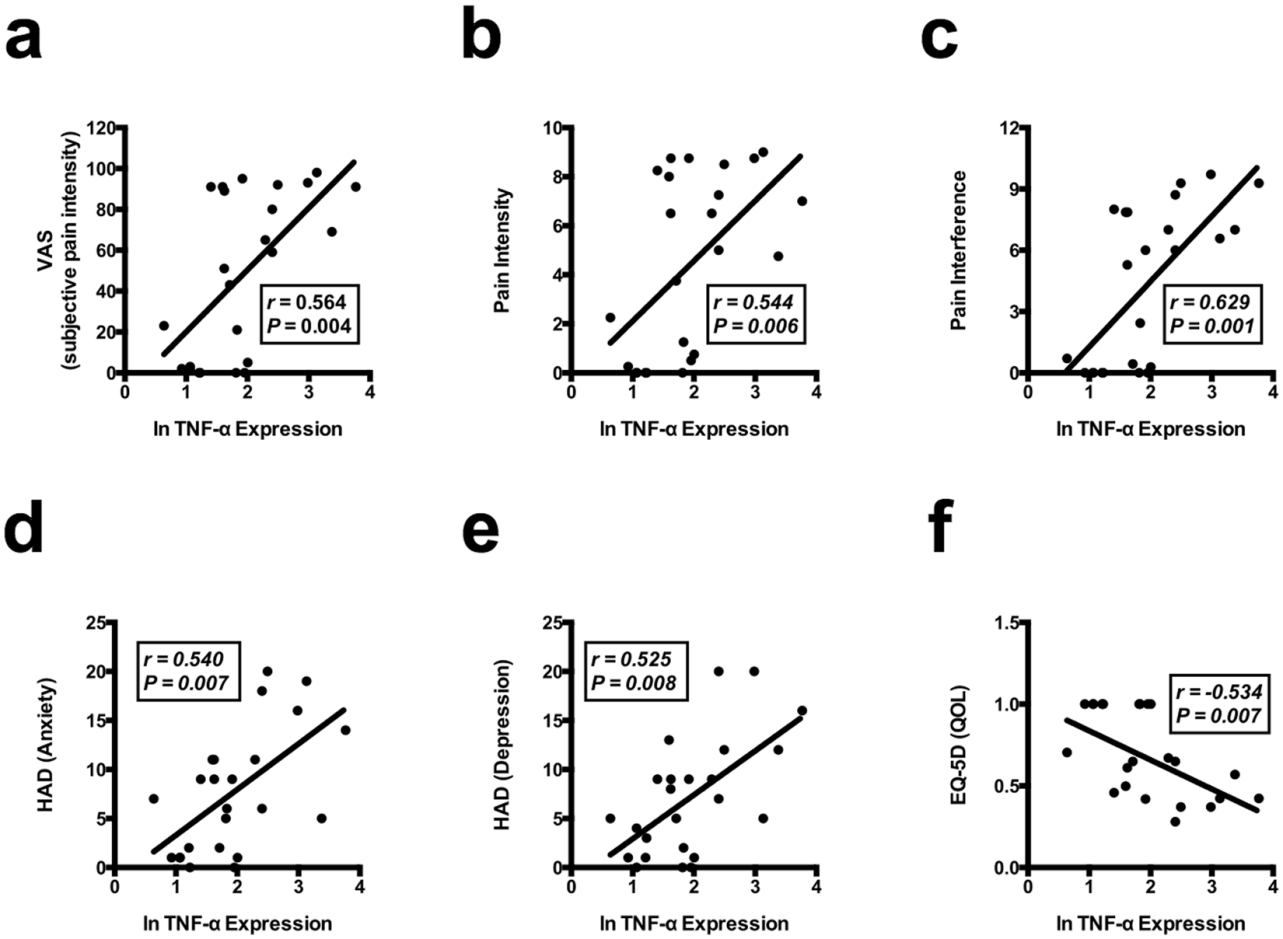
Correlation analyses between TNF-α expression and subjective clinical scores. Correlation between the natural log of fold-increase in TNF-α expression with iMG cells during ATP stimulation and several subjective clinical scores (**a**) SF-MPQ-VAS; (**b**) BPI-pain intensity; (**c**) BPI-pain interference; (**d**) HAD (anxiety); (**e**) HAD (depression); (**f**) EQ-5D (QOL). As a result of Shapiro-Wilk normality test, correlations were analyzed by the Spearman rank correlation test using the data of all subjects (healthy participants and patients with fibromyalgia). *r* indicates the correlation coefficient.

We conducted further correlational analyses between TNF-α gene expression in ATP-stimulated iMG cells and various clinical parameters including the severity of pain (Supplementary Table 2). Interestingly, we show a moderately positive correlation between TNF-α expression level and subjective pain intensity assessed by the visual analog scale (VAS) (Fig. 2a). Pain intensity and pain interference are also moderately positively correlated with TNF-α expression level (Fig. 2b,c). Fibromyalgia is a disease that is often comorbid with psychiatric disorders^20^. In particular, a relationship between fibromyalgia and psychiatric symptoms such as depression and anxiety has been reported in a previous study^21^. Here, we show a moderate positive correlation between TNF-α expression level and severity of both anxiety and depression (Fig. 2d,e). In contrast, IL-1β is not significantly correlated with any clinical scores (Supplementary Fig. 3). On the other hand, a moderate negative correlation is observed between TNF-α expression level and QOL (Fig. 2f). These findings suggest the possibility that the degree of subjective pain, psychiatric symptoms (depression and anxiety) and QOL in patients with fibromyalgia is controlled by the levels of microglia-derived TNF-α. Indeed, rodent studies have shown that TNF-α is an important factor in neuropathic pain^22,23^, and we previously demonstrated that TNF-α exhibits pain-related behaviors when administered into the lateral ventricle in rats^24^. TNF-α is also considered to play an important role in psychiatric disorders such as depression^25,26^. In addition, modulating microglia-derived TNF-α is suggested to be one of the therapeutic targets for psychiatric disorders^14,27,28^. Fibromyalgia is often comorbid with psychiatric disorders especially depression possibly due to shared pathological traits^20^.

This is the first study to suggest abnormal activation of microglia in fibromyalgia at the human cellular level. The present data indicate the positive relationship between microglial abnormality and clinical symptoms of fibromyalgia. Interestingly, iMG cells of fibromyalgia showed hyperresponsiveness to extracellular ATP and increases in the proinflammatory cytokine TNF-α. IL-1β is an important proinflammatory cytokine in the process of microglial activation via inflammasome signaling^29^. In the present study, gene expression of IL-1β (mRNA) is not changed, while the protein level of IL-1β in the supernatant is increased. These data suggest that microglial IL-1β is activated in earlier stages compared to TNF-α. Similar to iMG cells, PBMC-derived macrophages enhanced TNF-α expression by ATP stimulation. Therefore, enhanced TNF-α expression of patients’ iMG cells by ATP may not be specific to iMG cells, and further investigation should be conducted to compare the responses to ATP between iMG cells and PBMC-derived macrophages. On the other hand, fractalkine (CX3CL1) is also reported to be involved in chronic pain as well as ATP^30,31^. Thus, not only ATP but also fractalkine may also modulate iMG cells, and further studies are needed to clarify how fractalkine modulates iMG cells in patients with fibromyalgia. In addition, further investigations should clarify whether inflammasome related molecules such as IL-18 and surface expressions such as HLA-DR, CD80 and CD86 are modulated in iMG cells from patients with fibromyalgia. Of particular note, statistically moderate correlations are observed between ATP-induced TNF-α expression and various subjective parameters of pain, depression, anxiety, and QOL. In conclusion, microglia-derived TNF-α may be a possible key modulating factor of fibromyalgia, and future translational research is needed to establish a novel diagnostic system and therapeutic strategies against fibromyalgia. We could not validate the cause-effect relationship in the present study design, thus prospective studies are needed. For example, time-course assay at different severity levels of pain in the same patient can reveal whether iMG analysis can be utilized as a severity assessment tool. We believe that iMG technique sheds new light on clarifying dynamic molecular pathologies of microglia and on developing objective assessment tools in a variety of non-organic brain diseases, and further translational studies are warranted.

## Materials and Methods

### Subjects

The present study was conducted in accordance with the Declaration of Helsinki and was approved by the Ethics Committee of the Graduate School of Medical Sciences, Kyushu University. We recruited female adult patients who were diagnosed with fibromyalgia based on the 1990 American College of Rheumatology (ACR) classification criteria^32^ Female adult healthy volunteers were also recruited (Supplementary table 1). Healthy volunteers (as control group participants) had no history of fibromyalgia. There were no significant differences in age between the experimental and control groups. All participants provided written informed consent, after which we drew blood samples and administered several pain scales and other psychometric assessments.

Study participants completed the following four self-administered questionnaires. Sensory and affective components of pain were assessed via the short-form McGill Pain Questionnaire (SF-MPQ). Overall pain intensity was measured using the Visual Analog Scale (VAS) of the SF-MPQ^33,34^ and the Brief Pain Inventory (BPI). The BPI Interference Scale is a 7-item self-report measure that assesses the extent to which pain interferes with various components of functioning such as activity, mood, and sleep^35^. The Pain Catastrophizing Scale (PCS) assesses three aspects of catastrophic thinking regarding the pain experience: rumination, magnification, and helplessness^36,37^. The Hospital Anxiety and Depression Scale (HADS) is a widely used measure of anxiety and depression^38^. The EuroQol-5 Dimensions (EQ-5D) consists of 5 quality-of-life (QOL) domains: mobility, self-care, usual activities, pain or discomfort, and anxiety or depression. Scores on the EQ-5D range from 0.594 to 1.00 with a higher score indicating better health-related quality of life^39,40^.

### Induction of induced microglia-like (iMG) cells from human peripheral blood

Peripheral blood was collected using a heparinized tube from healthy volunteers and patients with fibromyalgia. Peripheral blood mononuclear cells (PBMCs) were isolated by Histopaque-1077 (Sigma Chemical Co., St. Louis, MO, USA) density gradient centrifugation. PBMCs were resuspended with RPMI-1640 (Nacalai Tesque, Kyoto, Japan), 10% heat-inactivated fetal bovine serum (FBS; Japan Bio Serum, Hiroshima, Japan), and 1% antibiotic/antimycotic (Invitrogen, Carlsbad, CA, USA). PBMCs were plated onto culture chambers at a density of 4 × 10^5^ cells/ml and cultured overnight in standard culture conditions (37 °C, 5% CO_2_). After overnight incubation, culture supernatant and non-adherent cells were removed. Adherent cells (monocytes) were cultured with RPMI-1640 Glutamax (Invitrogen) supplemented with 1% antibiotic/antimycotic and recombinant human GM-CSF (10 ng/ml; R&D Systems, Minneapolis, MN, USA) and recombinant human IL-34 (100 ng/ml; R&D Systems) for 14 days to develop the iMG cells^14,15^.

### Quantitative real time-polymerase chain reaction (qRT-PCR)

To assess gene expression patterns in iMG cells after treatment with adenosine triphosphate (ATP) or during phagocytosis, we performed qRT-PCR using a LightCycler 480 system (Roche Diagnostics, Mannheim, Germany). ATP (50 µg/ml; Sigma) or latex beads-FITC solution (Cayman Chemical) was added to the iMG cells and stimulated in standard culture conditions. After the stimulation by ATP (one hour) or beads (24 hours), the iMG cells were washed respectively, and total RNA was extracted using a High Pure RNA Isolation kit (Roche Diagnostics) according to the manufacturer’s protocol, and subjected to cDNA synthesis using a Transcriptor First Strand cDNA Synthesis kit (Roche Diagnostics). qRT-PCR for TNF-α and IL-β was performed using their respective primers. Primer sequences were as follows: TNF-α, F: 5′-CAGCCTCTTCTCCTTCCTGAT-3′, R: 5′-GCCAGAGGGCTFATTAGAGA-3′; IL-1β, F: 5′-TACCTGTCCTGCGTGTTGAA-3′, R: 5′-TCTTTGGGTAATTTTTGGGATCT-3′. Beta 2-microglobulin of the Universal ProbeLibrary (Roche Diagnostics) was used as a housekeeping control gene.

### Cytokine measurement

Cytokine (TNF-α, IL-1β, IL-6, IL-8 and IL-10) concentrations of blood plasma and culture supernatant of iMG cells during ATP stimulation were measured by the Cytometric Beads Array System (CBA; BD Biosciences, Franklin Lakes, NJ, USA) according to the manufacturer’s protocol. After incubation with ATP for one hour, culture supernatants were centrifuged at 10000 x g for 10 minutes and kept frozen at −80 °C until assayed. Plasma was also kept frozen at −80 °C until assayed. The culture supernatant and plasma were incubated with cytokine capture beads and PE-conjugated detection antibodies according to each protocol. After incubation, capture beads were washed and measurement data were acquired using a FACS ARIA™ flow cytometer (BD Biosciences). Data analysis was performed using FCAP Array software (BD Biosciences).

### Phagocytosis

Phagocytosis was examined via image-base cytometer (TALI, Invitrogen) using a Phagocytosis Assay Kit (Cayman Chemical, Ann Arbor, MI, USA) according to the manufacturer’s protocol. iMG cells were cultured in 24-well plates (Corning, Corning, NY, USA) at a density of 4 × 10^5^ cells/ml. We added 50 μl of the latex beads-FITC solution to each well, and incubated the cells in standard culture conditions for 24 hours. After harvesting the cells, we measured the fluorescence intensity of FITC using an image-base cytometer.

### Statistical analyses

Normality was assessed by Shapiro-Wilk normality tests. In the case of normal distribution, statistical differences between groups (healthy participants versus patients with fibromyalgia) were analyzed by Student’s *t*-test (two-tailed). In the case of non-normal distribution, statistical differences between groups were analyzed by Mann-Whitney *U* test (two-tailed). Correlations were analyzed by the Spearman rank correlation test using the data of all subjects (healthy participants and patients with fibromyalgia). In the correlation analysis, we used natural log of fold-increase in TNF-α expression with iMG cells during ATP stimulation. All statistical analyses were performed using GraphPad Prism 6 (GraphPad Software, Inc., La Jolla, CA, USA). *P* < 0.05 was considered statistically significant.

## Electronic supplementary material

Supplemental Dataset 1
