# Supplementary material for: Fibromyalgia and microglial TNF-α: Translational research using human blood induced microglia-like cells
Source: Sci Rep. 2017 Sep 19;7:11882. doi: 10.1038/s41598-017-11506-4 (PMC5605512; doi:10.1038/s41598-017-11506-4)
Supplement: Supplementary file 1 — Supplemental Dataset 1 [file 41598_2017_11506_MOESM1_ESM.doc]

**Supplementary Data**

**Fibromyalgia and microglial TNF-α: Translational research using human blood induced microglia-like cells.**

Masahiro Ohgidani PhD., Takahiro A. Kato M.D., PhD., Masako Hosoi M.D., PhD., Makoto Tsuda PhD., Kohei Hayakawa M.D., Chie Hayaki M.D, Rie Iwaki M.D., PhD., Noriaki Sagata PhD., Ryota Hashimoto M.D., PhD., Kazuhide Inoue PhD., Nobuyuki Sudo M.D., PhD., Shigenobu Kanba M.D., PhD.


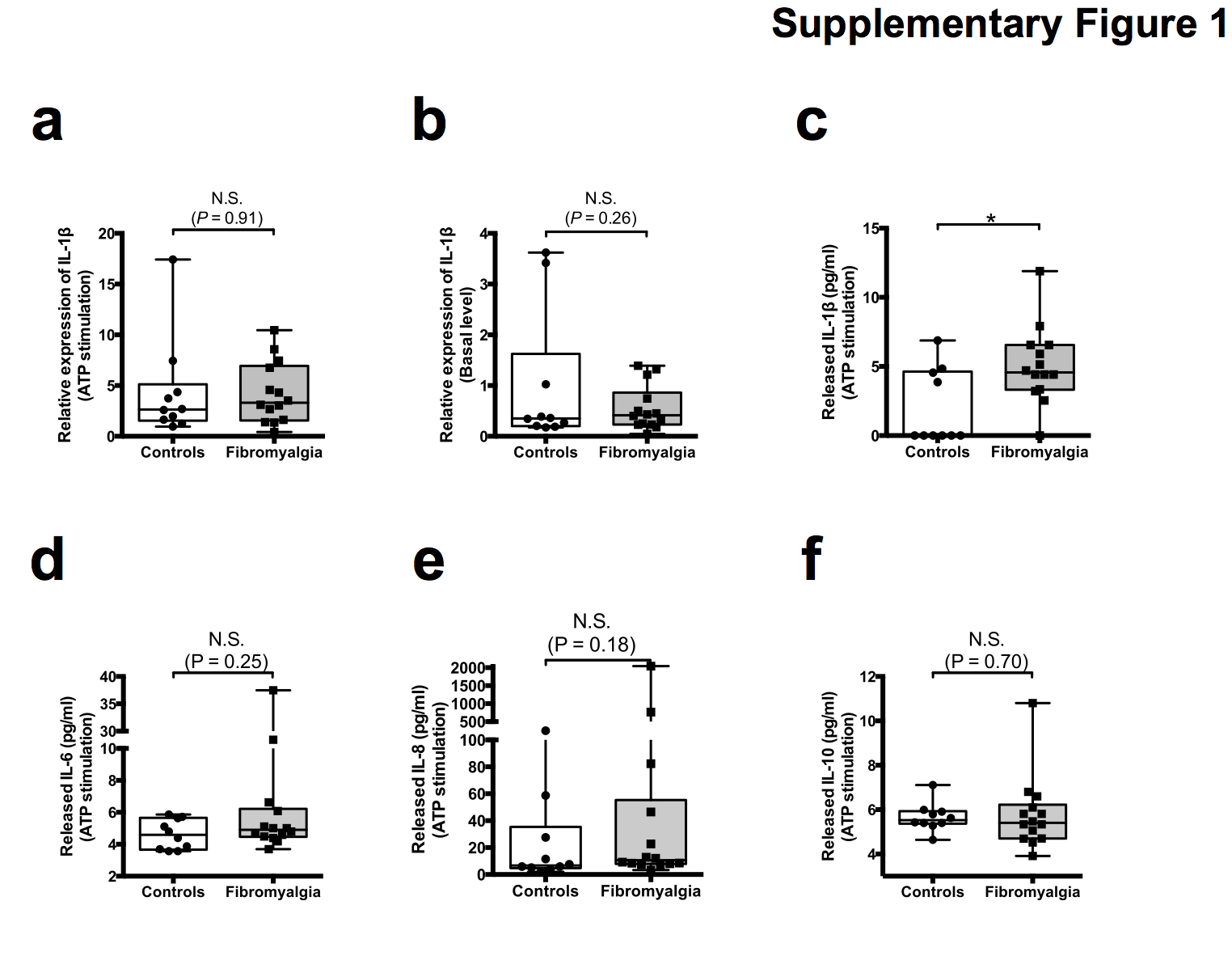
**Supplemental Figure 1. Gene expression and cytokine release during ATP stimulation.**

(a) Box-and-whisker plot showing gene expression of IL-1β during ATP stimulation in iMG cells from patients with fibromyalgia (n = 14; 25^th^ percentile, 1.56; mean, 4.23; 75^th^ percentile, 6.94) and healthy volunteers (n = 10; 25^th^ percentile, 1.55; mean, 4.41; 75^th^ percentile, 5.13). (b) Basal gene expression of IL-1β in iMG cells of patients with fibromyalgia (n = 14; 25^th^ percentile, 0.23; mean, 0.55; 75^th^ percentile, 0.86) and healthy volunteers (n = 10; 25^th^ percentile, 0.20; mean, 1.00; 75^th^ percentile, 1.62). (c) IL-1β concentration of the supernatant released during ATP stimulation by iMG cells from patients with fibromyalgia (n = 14; 25^th^ percentile, 3.32; mean, 5.07; 75^th^ percentile, 6.55) and healthy volunteers (n = 10; 25^th^ percentile, 0.00; mean, 2.02; 75^th^ percentile, 4.63). (d) IL-6 concentration of the supernatant released during ATP stimulation by iMG cells from patients with fibromyalgia (n = 14; 25^th^ percentile, 4.47; mean, 7.65; 75^th^ percentile, 6.22) and healthy volunteers (n = 10; 25^th^ percentile, 3.66; mean, 4.62; 75^th^ percentile, 5.66). (e) IL-8 concentration of the supernatant released during ATP stimulation by iMG cells from patients with fibromyalgia (n = 14; 25^th^ percentile, 7.88; mean, 216; 75^th^ percentile, 55.4) and healthy volunteers (n = 10; 25^th^ percentile, 4.80; mean, 42.4; 75^th^ percentile, 35.6). (f) IL-10 concentration of the supernatant released during ATP stimulation by iMG cells from patients with fibromyalgia (n = 14; 25^th^ percentile, 4.70; mean, 5.78; 75^th^ percentile, 6.23) and healthy volunteers (n = 10; 25^th^ percentile, 5.37; mean, 5.66; 75^th^ percentile, 5.93). The y-axis represents the expression levels for each group normalized by the data from a non-ATP treatment group (NT: iMG cells without ATP stimulation) (a) or normalized by healthy volunteers (b). As a result of Shapiro-Wilk normality test, statistical differences between groups were analyzed by Student’s *t*-test (two-tailed) (a and b) or Mann-Whitney *U* test (two-tailed) (c-f).


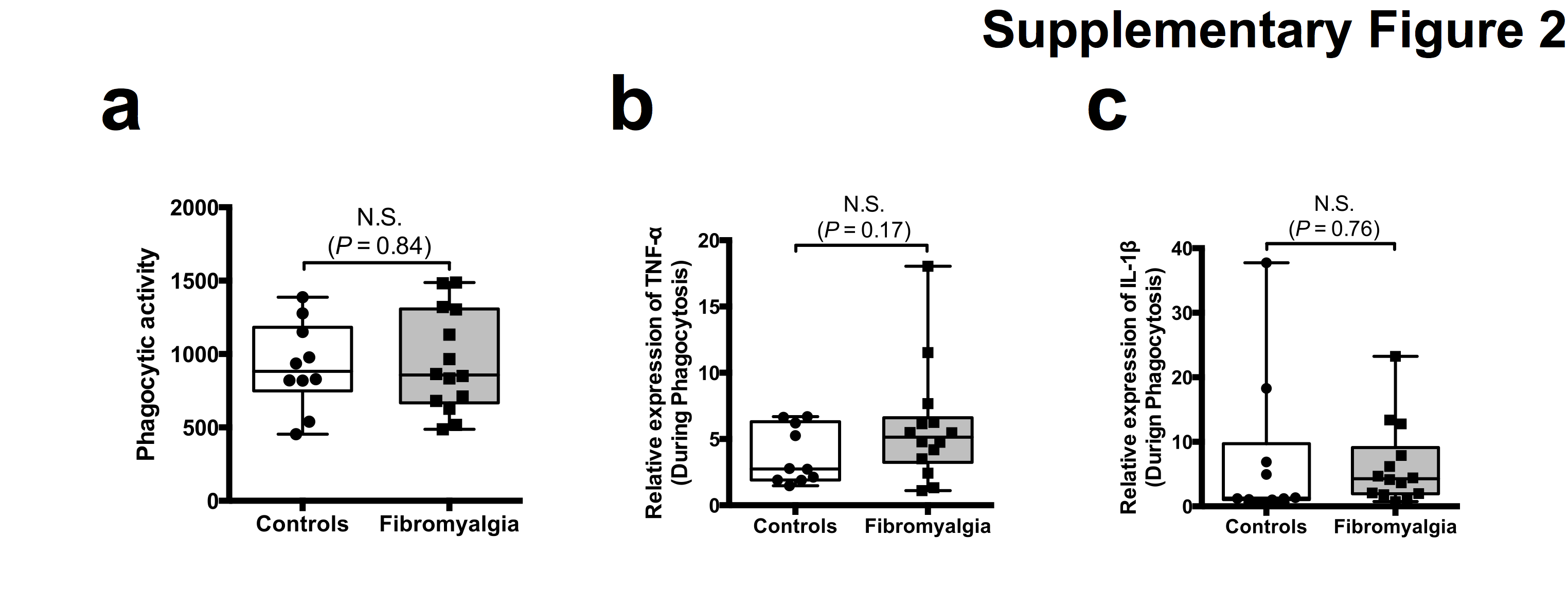
**Supplemental Figure 2. Phagocytic activity and gene expression of inflammatory cytokines during phagocytosis.**

(a) Box-and-whisker plot showing fluorescence intensity derived from phagocytosis (phagocytic activity) in iMG cells from patients with fibromyalgia (n = 14; 25^th^ percentile, 667.5; mean, 947.5; 75^th^ percentile, 1308.0) and healthy volunteers (n = 10; 25^th^ percentile, 749.0; mean, 919.2; 75^th^ percentile, 1183.0). (b) Gene expression of TNF-α during phagocytosis in iMG cells from patients with fibromyalgia (n = 14; 25^th^ percentile, 3.24; mean, 5.91; 75^th^ percentile, 6.61) and healthy volunteers (n = 10; 25^th^ percentile, 1.89; mean, 3.75; 75^th^ percentile, 6.30). (c) Gene expression of IL-1β during phagocytosis in iMG cells from patients with fibromyalgia (n = 14; 25^th^ percentile, 1.95; mean, 6.30; 75^th^ percentile, 9.12) and healthy volunteers (n = 10; 25^th^ percentile, 1.07; mean, 7.44; 75^th^ percentile, 9.73). The y-axis represents the expression levels for each group normalized by the data from a non-ATP treatment group (NT: iMG cells without beads) (b and c). As a result of Shapiro-Wilk normality test, statistical differences between groups were analyzed by Student’s *t*-test (two-tailed). N.S., not significant.


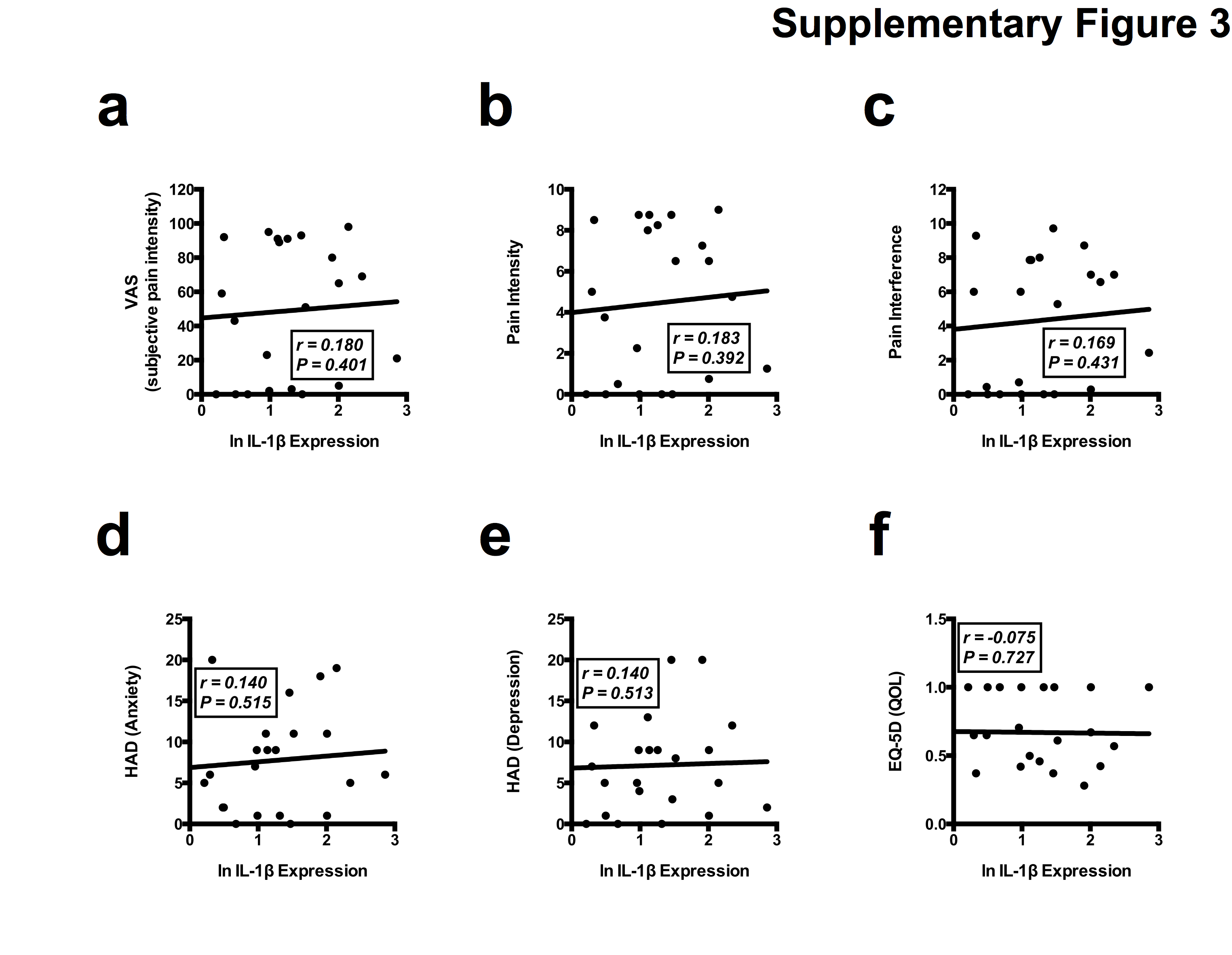
**Supplemental Figure 3. Correlation analyses between IL-1β expression and subjective clinical scores.** Correlation between the natural log of IL-1β expression in iMG cells from all subjects during ATP stimulation and several subjective clinical scores (a, SF-MPQ-VAS; b, BPI-pain intensity; c, BPI-pain interference; d, HAD-anxiety; e, HAD-depression; f, EQ-5D. As a result of Shapiro-Wilk normality test, correlations were analyzed by the Spearman rank correlation test. *r* indicates the correlation coefficient.

**Supplementary Table 1. Characteristics of the study participants.**

|  | **Control Group**  **(Healthy volunteers)** | **Patients with fibromyalgia** |
| --- | --- | --- |
| **Sample** | 10 | 14 |
| **Sex** | Female | Female |
| **Age (SD)^#^** | 40 ±10.3 | 40 ± 6.8 |
| **Pain duration (months)** | N/A | 87 (59-146) |

SD: standard deviation. The *P*-value was calculated by Student’s *t*-test (two-tailed).

Plus-minus values are means ± SD.

^#^P = 0.85

**Supplementary Table 2. Analysis of clinical pain scores for patients with fibromyalgia and healthy volunteers.**

| **Variable** | | **Control Group**  **(Healthy volunteers)** | **Patients with fibromyalgia** |
| --- | --- | --- | --- |
| **SF-MPQ** | |  |  |
|  | **VAS* (mm)** | 5.6 ± 8.8 | 79.1 ± 18.2 |
|  | **Sensory*** | 0.6 ± 1.1 | 24.7 ± 8.4 |
|  | **Affective*** | 0.2 ± 0.4 | 7.7 ± 4.3 |
|  | **Total*** | 0.8 ± 1.5 | 32.4 ± 12.4 |
| **BPI** | |  |  |
|  | **Pain intensity*** | 0.5 ± 0.8 | 7.2 ± 1.7 |
|  | **Pain interference*** | 0.3 ± 0.8 | 7.1 ± 2.3 |
| **PCS** | |  |  |
|  | **Total†** | 11.4 ± 11.8 | 33.8 ± 15.3 |
|  | **Rumination‡** | 7.3 ± 6.4 | 14.4 ± 5.8 |
|  | **Helplessness*** | 2.6 ± 4.0 | 11.9 ± 5.5 |
|  | **Magnification†** | 1.5 ± 2.2 | 7.5 ± 4.5 |
| **HAD** | |  |  |
|  | **Anxiety*** | 2.4 ± 2.6 | 11.4 ± 5.4 |
|  | **Depression*** | 1.7 ± 1.8 | 11.0 ± 4.9 |
| **EQ-5D*** | | 1.0 ± 0.1 | 0.5 ± 0.2 |

BPI, brief pain inventory; SF-MPQ, short-form McGill pain questionnaire; VAS, visual analog scale; PCS, pain catastrophizing scale; HAD, hospital anxiety depression scale; EQ-5D, EuroQol 5 dimension.

Plus-minus values are means ± SD.

*P*-value was calculated by Mann-Whitney *U* test (two-tailed). ******P* < 0.001, **†***P* < 0.01, ‡*P* < 0.05
